# Cognitive impairment profiles in patients with cerebrovascular diseases: a latent profile analysis

**DOI:** 10.3389/fneur.2026.1840409

**Published:** 2026-10-16

**Authors:** Yao Yin, Hui Ma, Rong Zou, Chaofeng Fan, Wei Zhu, Jun Zheng

**Affiliations:** 1 Department of Neurosurgery, West China Hospital, Sichuan University, Chengdu, China; 2 West China School of Nursing, Sichuan University, Chengdu, China; 3 Department of Rehabilitation Medicine, West China Hospital, Sichuan University, Chengdu, China; 4 Qinghai Blood Center, Xining, China

**Keywords:** associated factors, cerebrovascular diseases, cognitive function, cognitive impairment, latent profile analysis

## Abstract

**Background:**

Cognitive impairment is an increasingly recognized condition predominantly attributable to cerebrovascular diseases (CVDs). It substantially diminishes patients’ quality of life and imposes considerable burdens on caregivers and healthcare systems. However, how cognitive impairment manifest across multiple functional domains in individuals with CVDs remains poorly understood, often resulting in generalized care plans that fail to meet patients’ specific rehabilitation needs. This study aimed to identify latent profiles of cognitive impairment and their associated factors in patients with CVDs using latent profile analysis (LPA).

**Methods:**

This cross-sectional study was conducted using convenience sampling in a tertiary hospital in southwestern China from November 2023 to September 2025. LPA was employed to identify distinct cognitive impairment profiles among patients with CVDs. Multinomial logistic regression was subsequently performed to examine factors associated with profile categories.

**Results:**

The final three-profile model classified 474 participants into three groups: a mild impairment group (82.5%), a moderate impairment group (11.4%), and a severe impairment group (6.1%). The three profiles reflected increasing severity of cognitive impairment among patients with CVDs. Compared with the mild impairment group, higher NIHSS scores (OR = 1.222, 95 %CI = 1.126–1.326, *p* < 0.001) were associated with higher odds of membership in the moderate impairment profile. Male gender and lower NIHSS scores were associated with lower odds of membership in the severe impairment profile (all *p* < 0.05).

**Conclusion:**

In this predominantly hemorrhagic and neurosurgical cohort, the retained three-profile model represented different levels of MMSE-based global cognitive severity among patients with CVDs. NIHSS score and gender showed exploratory associations with profile membership. The applicability of these findings to broader CVD populations requires further validation.

## 1 Introduction

Cerebrovascular diseases (CVDs) remain a leading cause of death and loss of healthy life among Chinese residents, presenting a critical challenge to public health systems (1). Despite advances in acute management, the burden of CVDs continues to rise, with a reported crude mortality rate of 149.6 per 100,000 individuals (2). Beyond physical impairments, cognitive impairment has emerged as a pervasive and debilitating complication, encompassing a spectrum from mild deficits to dementia (3, 4). The prognostic implications of cognitive impairment are profound, evidence suggests that even among individuals adhering to optimal lifestyle behaviors, the presence of cognitive impairment significantly elevates the risk of all-cause and cerebrovascular mortality (5). High prevalence rates have been reported across various CVD subtypes, including stroke (66%) and moyamoya disease (54.59%), severely compromising patients’ quality of life and increasing the burden on healthcare resources (6, 7).

Traditionally, clinical and nursing research on cognitive impairment has predominantly followed a “variable-centered” research path. This methodology relies on global cognitive screening instruments, such as the Mini-Mental State Examination (MMSE) or the Montreal Cognitive Assessment (MoCA), to categorize patients via dichotomous cut-off scores (8, 9). While effective for identifying universal risk factors (e.g., age, comorbidities, or lesion sites), this approach assumes population homogeneity. It fails to account for latent heterogeneity or distinct patient subgroups, as it cannot simultaneously analyze individual performance across multiple cognitive domains (e.g., memory, orientation, and language ability). Consequently, such generalized metrics may lead to “one-size-fits-all” care plans that overlook the specific rehabilitation needs of diverse patient profiles.

To address these limitations, an “individual-centered” analytical approach is warranted. Latent Profile Analysis (LPA) offers a sophisticated statistical alternative by identifying heterogeneity among individuals (latent profiles) within a population based on response patterns across multiple continuous indicators (28). Recent studies have employed LPA to identify cognitive impairment profile categories in older adults, stroke survivors, and individuals with cardiometabolic diseases (10–12). These investigations consistently identified three cognition profiles based on MMSE performance: a high-performing group, a severely impaired group, and an intermediate group characterized by domain-specific deficits. Notably, the distinguishing features of the intermediate profile varied across disease populations, suggesting disease-specific patterns of cognitive compromise. However, research applying LPA to characterize cognitive impairment categories targeting the broader CVDs population remains scarce.

Therefore, this study aimed to identify the latent profiles of cognitive impairment in patients with CVDs and explore their associated factors. By characterizing different levels in global cognitive impairment severity among patients with CVDs, this study may support the management of cognitive impairment among patients with CVDs and the development of effective interventions.

## 2 Materials and methods

### 2.1 Study design, setting and participants

The study was prepared and reported according to the Strengthening the Reporting of Observational Studies in Epidemiology (STROBE) checklists to enhance transparency and mitigate reporting bias.

This cross-sectional study was conducted using convenience sampling in a tertiary hospital in southwestern China from November 2023 to September 2025. This hospital serves as a leading tertiary referral center for the management of diverse CVDs, including AVMs, intracranial aneurysms, stroke, et al. All research participants were recruited from the hospital and had a primary diagnosis of CVDs. The inclusion criteria were: (1) age 18 years or older; (2) diagnosis of CVDs via ICD-10 codes (I60-I68), and confirmed without misdiagnosis by cranial CT or MRI; (3) volunteer to participate and signed informed consent by themselves or their families. Exclusion criteria included: (1) had a previously established diagnosis of cognitive impairment or dementia attributable to other medical conditions; (2) a history of severe auditory impairment, psychiatric illness or communication disorders; (3) acute critical medical conditions (e.g., shock, respiratory failure, acute heart failure, acute myocardial infarction, or stroke); (4) were concurrently enrolled in other clinical trials.

### 2.2 Sample size estimation

Although there is currently no sample formula or calculator to estimate required sample size in the LPA, some simulation studies have suggested samples of 300 to 500 would qualify as a minimum sample (13). And LPA was considered a ‘large sample’ method. Models and fit statistics have been shown to consistently perform with low reliability, when the sample size was less than 300 (14). Ultimately, the sample size of this study was 474 participants.

### 2.3 Data collection

Data were collected by research assistants in the outpatient departments from patients who were treated at the study hospital with a primary diagnosis of CVDs. These assistants were employed under the supervision of specialized physicians in CVDs at the hospital. Prior to data collection, all research assistants underwent comprehensive training on the study design, objectives, procedures, and measurement protocols. Informed consent was obtained from each participant before data collection began. Following this, the data were carefully reviewed for accuracy.

### 2.4 Measures

#### 2.4.1 Demographic and disease-related characteristics

The demographic and disease-related characteristics included in the questionnaire were determined by the research team following a comprehensive review of the relevant literature and subsequent discussions. Demographic variables encompassed age, body mass index (BMI), gender, ethnicity, educational background, marital status, residence, employment status, and a history of smoking and alcohol consumption. Disease-related variables included disease subtypes, comorbidities, follow-up interval (discharge-to-follow-up interval), surgery, use of analgesics, sedation, and mechanical ventilation, as well as the Nutritional Risk Screening 2002 (NRS-2002) score, and the length of hospital stay.

#### 2.4.2 The Chinese Mini-Mental State Examination (MMSE)

The MMSE has been widely used for screening for cognitive function for patients, which was developed by Folstein et al. (15). The tool consists of 30 items, assessing five dimensions of orientation, memory, attention and calculation, recall and language ability. The total score ranges from 0 to 30. Higher scores indicate less impairment of cognitive function. According to educational level, cognitive impairment was defined as MMSE scores < 24 with education ≥ 7 years or < 20 with education < 7 years. The Chinese version of the MMSE translated and adopted by Zhang et al. [17]is widely utilized in clinical screening for patients with cerebrovascular disease-related cognitive impairment in China. The Cronbach’s α was 0.83 (16).

#### 2.4.3 National Institutes of Health Stroke Scale (NIHSS)

It is primarily used to assess the severity and neurological function of stroke but has also been extended to evaluate other cerebrovascular conditions, such as AVMs and MMD, as recommended by Chinese clinical practice guidelines (17, 18). Healthcare providers use the NIHSS to measure neurological function and deficits through a series of 11 questions and both physical and mental assessments. The score ranges from 0, indicating no symptoms, to 42, indicating severe stroke, with higher scores correlating with increased disease severity.

### 2.5 Statistical analysis

Data were analyzed using IBM SPSS version 29.0. Descriptive statistics were used to characterize patient demographics, with continuous variables reported as means ± standard deviations (SD) or medians (interquartile range), and categorical variables as frequencies and proportions. Kruskal-Wallis and chi-squared tests were applied to compare demographic and disease-related variables across subgroups. The statistically significant variables were further analyzed using multinomial logistic regression. A significance level of *p* < 0.05 was used for all analyses.

Demographic adjustment and LPA were conducted using R version 4.4.0 (R Foundation for Statistical Computing, Vienna, Austria). The five MMSE domain scores were separately adjusted for age, gender, and years of education using ordinary least-squares linear regression, implemented with the *lm()* function in the *stats* package. These demographically adjusted residual z-scores were subsequently used as indicators in the LPA performed using the *tidyLPA* package (19). The complete annotated analysis code has been deposited in Zenodo and is publicly available at [DOI: https://doi.org/10.5281/zenodo.22998558]. The number of classes was incrementally increased until model fit indices no longer improved. Model selection was based on the Akaike Information Criterion (AIC), Bayesian Information Criterion (BIC), and Adjusted BIC, with lower values indicating better fit. Entropy values closer to 1 indicated more precise classification. The Lo–Mendell–Rubin likelihood ratio (LMR) and Bootstrapped Likelihood Ratio Tests (BLRT) were used to compare model fits, with a *p*-value < 0.05 indicating a better k-class model than a (k-1)-class model. Selection of the retained solution was informed by statistical fit, parsimony, and substantive interpretability (20). These considerations were evaluated in relation to the objective of describing broad levels of cognitive impairment severity.

### 2.6 Ethical considerations

After receiving a comprehensive description of the study’s objectives and procedures, each participant gave signed informed consent. Throughout the investigation, no personally identifiable information about the patients was revealed. The Declaration of Helsinki and Good Clinical Practice principles were followed in the conduct of the study. This study was approved by the West China Hospital of the Sichuan University Biomedical Research Ethics Committee (no.888[2022]).

## 3 Results

### 3.1 Demographic and disease-related characteristics

As shown in Table 1, in this cohort of 474 patients with cerebrovascular disease, the median age was 52 years (ranging 18–84), with a near-equal gender distribution (49.6% male) and a majority of Han ethnicity (91.8%). Clinically, intracerebral hemorrhage was the most prevalent diagnosis (52.3%), and hypertension was a common comorbidity (45.0%). A key characteristic of this population was the high acuity of care required, as evidenced by the fact that the majority underwent surgical intervention (79.7%) and received intensive supportive management, including analgesia (80.1%), sedation (73.2%), and mechanical ventilation (67.0%). With this high level of intervention, the median NIHSS score was 0 (range 0–26), indicating minimal neurological deficits at assessment for a substantial portion of the cohort, and the median hospital length of stay was 9 days (IQR 6–12).

**Table 1:** Demographic and disease-related characteristics of participants(n = 474).

Variables	Category	Median (IQR)/N (%)
Age, y		52 (41, 59)
BMI		24 (22, 26)
Gender	Male	235 (49.6)
	Female	239 (50.4)
Ethnicity	Han	434 (91.8)
	Minority	39 (8.2)
Educational background, y		9 (9, 15)
Marital status	Married	399 (84.2)
	Single/Divorced/Widowed	75 (15.8)
Residence	Urban	327 (69.1)
	Rural	146 (30.9)
Employment status	Employment	137 (28.9)
	Unemployment/Unknown	337 (71.1)
Smoking	Yes	78 (16.5)
Alcohol consumption	Yes	69 (14.6)
Subtypes of disease	Intracerebral hemorrhage	248 (52.3)
	Ischemic stroke	22 (4.6)
	AVMs	28 (5.9)
	Intracranial aneurysms	71 (15.0)
	MMD	89 (18.8)
	Other	16 (3.4)
Comorbidities	Hypertension	213 (45.0)
	Diabetes	48 (10.1)
	Coronary heart disease	10 (2.1)
	Hyperlipidemia	35 (7.4)
Diseases duration, d		14 (7, 108)
Underwent surgery	Yes	378 (79.7)
History of analgesic use	Yes	379 (80.1)
History of sedative use	Yes	346 (73.2)
History of receiving mechanical ventilation	Yes	317 (67.0)
NRS-2002		1 (0, 3)
NIHSS score		0 (0, 3)
Length of hospital stay, d		9 (6, 12)

IQR, Interquartile range; BMI, Body mass index; AVMs, Arteriovenous malformations; MMD, Moyamoya disease; NRS-2002, Nutritional risk screen-2002; NIHSS, National Institutes of Health Stroke Scale.

### 3.2 Scores of MMSE dimensions among patients with CVDs

The cognitive profile of the cohort, as assessed by the MMSE, revealed a mean total score of 22.92 ± 7.25, indicating overall mild cognitive impairment. Analysis of the specific domains showed notable variations. The most significant deficits were observed in recall (mean 1.66 ± 1.20 out of 3) and orientation (mean 7.72 ± 2.79 out of 10). In contrast, memory (registration) and language ability were relatively better preserved, with mean scores of 2.75 (±0.75) out of 3 and 7.79 (±2.06) out of 9, respectively. Performance in attention and calculation was moderately affected (3.01 ± 1.93 out of 5). Detailed information was shown in Table 2.

**Table 2:** Scores of MMSE dimensions among patients with CVDs (*n* = 474).

Dimensions	Number of items	Score range	Scores (Means ± SD)
Orientation	10	0–10	7.72 ± 2.79
Recall	3	0–3	1.66 ±1.20
Attention and calculation	5	0–5	3.01 ±1.93
Memory	3	0–3	2.75 ± 0.75
Language ability	9	0–9	7.79 ±2.06
Total score	30	0–30	22.92 ±7.25

SD, standard deviation.

### 3.3 Latent profile analysis of cognitive impairment in patients with CVDs

The five demographically adjusted residual z scores were entered as continuous indicators in an LPA to identify profiles representing different levels of global cognitive impairment severity. One- through five-profile solutions were estimated (Table 3). AIC, BIC, and aBIC decreased as profiles were added, and the LMR and BLRT comparisons were significant for the two- through five-profile solutions (all *p* < 0.001). Thus, the four- and five-profile solutions showed better statistical fit than the three-profile solution according to these criteria. However, the three-profile model showed greater classification certainty (entropy = 0.955) than the four- and five-profile models (0.859 and 0.815, respectively). We retained the three-profile model to provide a parsimonious and clinically interpretable description of broad differences in cognitive impairment severity within the cohort.

**Table 3:** Fit indices for latent profile models of cognitive impairment in patients with CVDs.

Model	LL	FP	AIC	BIC	aBIC	Entropy	LMR (*P*)	BLRT (*P*)	Probability of class (%)
1	−3360.382	10	6740.763	6782.375	6750.637	-	-	-	100.0
2	−2686.641	16	5405.281	5471.861	5421.079	0.997	<0.001	<0.001	92.2/7.8
3 †	−2507.467	22	5058.935	5150.481	5080.657	0.955	<0.001	<0.001	82.5/11.4/6.1
4	−2477.727	28	5011.454	5127.968	5039.100	0.859	<0.001	<0.001	57.4/9.7/26.8/6.1
5	−2443.929	34	4955.858	5097.339	4989.428	0.815	<0.001	<0.001	44.5/9.5/25.3/14.6/6.1

†The three-profile model was retained for subsequent analyses as a pragmatic choice based on parsimony and clinical interpretability. Profile-specific indicator means were freely estimated, indicator-specific variances were constrained to be equal across profiles, and within-profile covariances were fixed at zero. Candidate models were first fitted using the default mclust initialization. Local-solution sensitivity was examined using 1,000 randomized hierarchical initializations for each two- through five-profile model. The BLRT was based on 1,000 bootstrap draws.

LL, log-likelihood; FP, free parameters; AIC, Akaike information criterion; BIC, Bayesian information criterion; aBIC, sample-size adjusted BIC; LMR, Lo–Mendell–Rubin likelihood ratio; BLRT, bootstrap likelihood ratio test.

### 3.4 Cognitive impairment severity profiles

Following LPA of the five demographically adjusted MMSE domain z-scores, we retained a three-profile model to describe differences in global cognitive impairment severity within the cohort (Figure 1). The largest profile was the “mild impairment” group (Class 1; 82.5%, n = 391). Participants in this group performed relatively well across all five cognitive domains. The “moderate impairment” group (Class 2; 11.4%, n = 54) showed lower performance across all domains, with the greatest deficits in orientation and attention/calculation, while memory was relatively preserved. The smallest profile was the “severe impairment” group (Class 3; 6.1%, n = 29), which was characterized by pervasive deficits across all domains. Overall, the three profiles reflected increasing severity of cognitive impairment among patients with CVDs.

**Figure 1:**
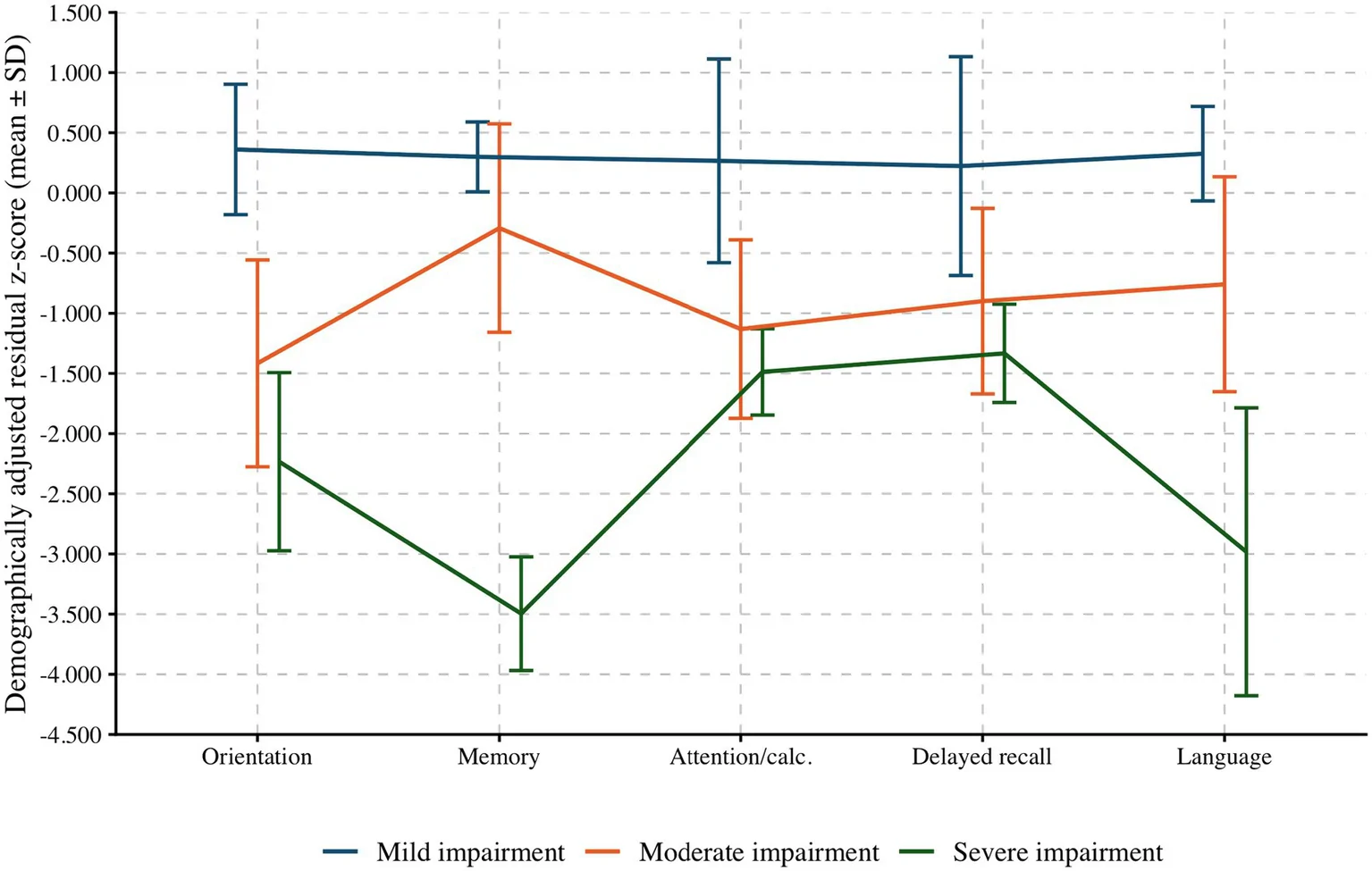
Cognitive impairment severity profiles based on demographically adjusted MMSE domain scores. Lines represent mean residual z-scores adjusted for age, gender, and years of education; error bars indicate ±1 SD. MMSE, Mini-Mental State Examination; SD, standard deviation.

### 3.5 Comparison of baseline information and univariate analysis across cognitive impairment profiles

Patient demographic and disease-related characteristics were used as the independent variable and three-class cognitive impairment profiles were used as the dependent variable. Univariate analyses revealed significant associations between the derived cognitive profiles and key demographic and clinical characteristics.

The three classes were demonstrated statistically significant differences in gender, educational background, alcohol consumption, subtypes of disease, follow-up interval, nutritional risk (NRS-2002), disease severity (NIHSS score) and length of hospital stay (all *p* < 0.05; refer to Table 4 for detailed results).

**Table 4:** Univariate analysis to explore inter-profile differences.

Variables	Category	Class 1 (*n* = 391)	Class 2 (*n* = 54)	Class 3 (*n* = 29)	(χ^²^/H)	*P*
Age, y		52 (40,59)	51 (42.61)	56 (48.62)	3.816	0.145
BMI		24 (22, 27)	24 (22.26)	23 (21,25)	2.675	0.262
Gender	Male	189 (48.3)	37 (68.5)	9 (31.0)	11.979	0.003
	Female	202 (51.7)	17 (31.5)	20 (69.0)		
Ethnicity	Han	355 (91.0)	51 (94.4)	28 (96.6)	-	0.589*
	Minority	35 (9.0)	3 (5.6)	1 (3.4)		
Educational background, y		12 (9,15)	9 (9.15)	9 (6.12)	7.412	0.025
Marital status	Married	334 (85.4)	40 (74.1)	25 (86.2)	4.683	0.096
	Single/divorced/widowed	57 (14.6)	14 (25.9)	4 (13.8)		
Residence	Urban	275 (70.5)	36 (66.7)	16 (55.2)	3.151	0.207
	Rural	115 (29.5)	18 (33.3)	13 (44.8)		
Employment status	Employment	117 (29.9)	16 (29.6)	4 (13.8)	3.434	0.180
	Unemployment/unknown	274 (70.1)	38 (70.4)	25 (86.2)		
Smoking	Yes	61 (15.6)	12 (22.2)	5 (17.2)	1.504	0.471
Alcohol consumption	Yes	51 (13.1)	14 (25.9)	4 (13.8)	6.301	0.043
Subtypes of disease	Intracerebral hemorrhage	187 (47.8)	38 (70.4)	23 (79.3)	-	0.013*
	Ischemic stroke	18 (4.6)	3 (5.6)	1 (3.4)		
	AVMs	24 (6.1)	3 (5.6)	1 (3.4)		
	Intracranial aneurysms	66 (16.9)	4 (7.4)	1 (3.4)		
	MMD	82 (21.0)	4 (7.4)	3 (10.3)		
	Other	14 (3.6)	2 (3.7)	0 (0.0)		
Comorbidities	Hypertension	172 (44.1)	27 (50.0)	14 (48.3)	0.798	0.671
	Diabetes	37 (9.5)	8 (14.8)	3 (10.3)	1.478	0.478
	Coronary heart disease	8 (2.1)	0 (0.0)	2 (6.9)	-	0.122*
	Hyperlipidemia	31 (7.9)	4 (7.4)	0 (0.0)	-	0.353*
Follow-up interval, d		1 (0,103)	0 (0.1)	0 (0.1)	21.542	<0.001
Underwent surgery	Yes	315 (80.6)	44 (81.5)	19 (65.5)	3.897	0.142
History administration of analgesia	Yes	311 (79.7)	44 (81.5)	24 (82.8)	0.224	0.894
History administration of sedation	Yes	282 (72.3)	41 (75.9)	23 (79.3)	0.913	0.633
History of receiving mechanical ventilation	Yes	259 (66.4)	37 (68.5)	21 (72.4)	0.502	0.778
NRS-2002		0 (0.2)	2 (2.3)	2 (2.5)	27.366	<0.001
NIHSS score		0 (0.2)	3 (1.7)	9 (4.15)	104.586	<0.001
Length of hospital stay, *d*		9 (6.11)	9 (6.5, 12)	12 (7.17)	6.213	0.045
MMSE score		27 (23.29)	15 (12.16)	3 (0.5)	195.720	<0.001

*Fisher’s exact test.

IQR, Interquartile range; BMI, Body mass index; AVMs, Arteriovenous malformations; MMD, Moyamoya disease; NRS-2002, Nutritional risk screen-2002; NIHSS, National Institutes of Health Stroke Scale; MMSE, the Chinese Mini-Mental State Examination.

### 3.6 Multivariate analysis of cognitive impairment profiles in patients with CVDs

To identify factors associated with different classes of cognitive impairment profile, a multinomial logistic regression analysis was performed. The model included variables that were statistically significant in univariate analyses or deemed clinically important, with the “mild impairment group” group designated as the reference category.

The multinomial logistic regression model, using the “mild impairment group” as the reference, identified several factors independently associated with membership in the “moderate impairment group “and “severe impairment group” (Table 5). Higher NIHSS scores (OR = 1.222, 95 %CI = 1.126–1.326) were associated with higher odds of membership in the moderate impairment group. Male gender (OR = 0.218, 95 %CI = 0.067–0.704) was associated with lower odds of membership in the severe-impairment profile, whereas higher NIHSS scores (OR = 1.397, 95 %CI = 1.254–1.557) were associated with higher odds of membership in this profile (see Table 5).

**Table 5:** Multinomial logistic regression analysis of latent cognitive impairment profiles in patients with CVDs.

Independent variables	Class 2			Class 3		
	*P*	OR	95%CI	*P*	OR	95%CI
Age, y	0.761	0.996	0.970–1.022	0.675	1.008	0.970–1.047
Gender (ref: female)						
Male	0.420	1.351	0.650–2.809	0.011	0.218	0.067–0.704
Educational background, y	0.233	0.951	0.875–1.033	0.112	0.902	0.794–1.024
Alcohol consumption (ref: yes)	0.097	0.511	0.231–1.129	0.240	0.422	0.100–1.780
Subtypes of disease (ref: intracerebral hemorrhage)						
Ischemic stroke	0.954	1.042	0.254–4.271	0.959	0.942	0.095–9.331
AVMs	0.823	1.172	0.292–4.710	0.637	0.583	0.062–5.513
Intracranial aneurysms	0.371	1.760	0.510–6.079	0.228	4.111	0.412–40.988
MMD	0.318	1.863	0.550–6.313	0.539	0.604	0.121–3.009
Other	-	-	-	-	-	-
Follow-up interval, d	0.062	0.996	0.992–1.000	0.359	0.997	0.992–1.003
NRS-2002	0.934	1.009	0.819–1.243	0.535	1.103	0.810–1.502
NIHSS score	<0.001	1.222	1.126–1.326	<0.001	1.397	1.254–1.557
Length of hospital stay, d	0.452	0.983	0.942–1.027	0.980	0.999	0.943–1.059

OR, Odds ratio; CI, Confidence interval; AVMs, Arteriovenous malformations; NRS-2002, MMD, Moyamoya disease; Nutritional risk screen-2002; NIHSS, National Institutes of Health Stroke Scale.

Multinomial logistic regression analysis of factors associated with moderate (Class 2) and severe impairment (Class 3) profiles (reference = mild impairment group).

## 4 Discussion

This study used LPA to characterize heterogeneity in cognitive impairment among patients with CVDs. We retained a three-profile model to describe broad differences in global cognitive impairment severity and examined factors associated with profile membership. The largest profile in the final three-profile model was the “mild impairment” group (Class 1; 82.5%, n = 391). Participants in this group performed relatively well across all five cognitive domains. The “moderate impairment” group (Class 2; 11.4%, n = 54) showed lower performance across all domains, with the greatest deficits in orientation and attention/calculation, while memory was relatively preserved. The smallest profile was the “severe impairment” group (Class 3; 6.1%, n = 29), which was characterized by pervasive deficits across all domains. Overall, the three profiles reflected increasing severity of cognitive impairment among patients with CVDs. It is important to note that these class proportions reflect the distribution within the present sample and are not intended to represent population prevalence. The emergence of these distinct profiles underscores the multidimensional nature of cognitive impairment captured by the MMSE, challenging the notion of a unitary, linear trajectory of cognitive decline and instead indicating the existence of specific-subgroup variations with potential implications for targeted nursing assessment and intervention.

Male participants exhibited statistically significant lower odds of severe cognitive impairment compared to females (OR = 0.218, *p* = 0.011). These findings align with previous research demonstrating sex differences in cognitive trajectories following cerebrovascular disease (21, 22), particularly the increased vulnerability of older women to rapid cognitive decline (10). This disparity may be mediated by physiological mechanisms involving sex steroid hormones. Females typically demonstrate higher baseline cerebral blood flow (CBF) than males (23), potentially rendering them more sensitive to hemodynamic fluctuations following cerebrovascular events. Furthermore, while sex hormones confer neuroprotection in younger physiology, preclinical evidence suggests these effects may become ineffective or even deleterious with advancing age (24). Notably, 64.0% (153/239) of the female participants in the present study were aged over 50 years. Additionally, the observed gender disparity may be influenced by sociodemographic factors, as women had significantly lower levels of educational attainment compared to men (9.85 ± 4.41 vs. 11.62 ± 4.11, *p* < 0.001). Further studies could assess biological or social mechanisms that might underlie this association.

Greater neurological deficit severity, as reflected by higher NIHSS scores, was associated with significantly higher odds of being classified into the moderate and severe impairment groups (OR = 1.222 and 1.397, respectively; both *p* < 0.001, reference group = the mild impairment group). This strong association aligns with the well-established prognostic utility of the NIHSS score in predicting cognitive outcomes following some CVDs (11). The effect of stroke severity and burden on the risk of dementia was evident in all age groups, with severe stroke (NIHSS score >10) bringing forward dementia by about 25 years (25). Mechanistically, a higher NIHSS score typically correlates with a larger infarct or hematoma volume and a lesion in a more critical location (e.g., lesions affecting the dominant hemisphere or key cognitive networks) (1). This greater initial tissue damage depletes the brain’s cognitive reserve, leaving the patient less capable of compensating for subsequent neuronal loss. However, these possible mechanisms could not be examined in our study because information on lesion volume, white matter hyperintensities, brain atrophy, and blood or cerebrospinal fluid biomarkers was not available. These unmeasured factors may have contributed to the observed differences in cognitive severity and may have resulted in residual confounding. The American Heart Association/American Stroke Association scientific statement also notes that post-stroke cognition may be affected by noncerebral factors, including infection, frailty, and social conditions, whose independent roles remain unclear (26). Future studies should combine assessments with neuroimaging, fluid biomarkers, and measures of infection, frailty, rehabilitation, and social conditions to identify independent predictors of cognitive impairment severity levels.

The nonsignificant estimates for other covariates should not be taken as evidence that these factors have no clinical relevance. The small number of participants in the severe-impairment profile (n = 29), corresponding to a category-specific events-per-parameter ratio of 2.4, limited the statistical precision of this comparison and resulted in wide confidence intervals (27). In addition, we adjusted for the potential influence of some of these variables in our analyses by using demographically (age, gender, and years of education) adjusted z-scores. The nonsignificant estimates for these demographic variables in the multinomial logistic regression should not be interpreted as evidence that they are unrelated to cognitive impairment.

## 5 Limitations

This study provides valuable insights into the heterogeneity of cognitive impairment in patients with CVDs; however, several limitations should be acknowledged. Participants were recruited from the neurosurgery department of a tertiary referral hospital. The cohort consisted mainly of patients with intracerebral hemorrhage (52.3%), moyamoya disease (18.8%), and intracranial aneurysms (15.0%), whereas ischemic stroke accounted for only 4.6%. This may introduce a selection bias, potentially limiting the generalizability of the identified latent cognitive profiles to different healthcare settings or patient populations. The cross-sectional design of the study inherently precludes causal inferences and does not allow for the characterization of individual cognitive trajectories over time. While the overall sample size was robust, the subgroup of patients exhibiting severe cognitive impairment had a relatively small sample size. This limitation may affect the stability and precision of the latent profile estimates for this specific group and could lead to reduced statistical power in subsequent analyses comparing this profile to others. Finally, the MMSE is a brief screening tool with limited coverage of executive function, processing speed, visuospatial ability, and episodic memory. Although the five MMSE domain scores were separately adjusted for age, gender, and years of education before the LPA, this adjustment cannot recover information lost because of these measurement limitations. Future multicenter, longitudinal studies with larger, more diverse samples are warranted to validate the current latent cognitive profiles and assess their prognostic utility.

## 6 Conclusion

The retained three-profile model provided a pragmatic summary of cognitive impairment severity among patients with CVDs: a mild impairment group (82.5%), a moderate impairment group (11.4%), and a severe impairment group (6.1%). The three profiles reflected increasing severity of cognitive impairment among patients with CVDs. Compared with the mild impairment group, male gender was associated with lower odds of membership in the severe-impairment profile. Higher NIHSS scores were associated with higher odds of membership in the moderate and severe impairment profile. Given the predominance of hemorrhagic and neurosurgical cases in this cohort, the generalizability of these findings to broader CVDs populations requires further validation.

## Data Availability

The raw data supporting the conclusions of this article will be made available by the authors, without undue reservation.
